# Prevalence of occupational injuries among construction workers in Karachi, Pakistan

**DOI:** 10.1371/journal.pgph.0004578

**Published:** 2025-04-29

**Authors:** Asad Allana, Aamir Ali Khan, Muhammad Yousuf, Paul Cullinan, Asaad Ahmed Nafees

**Affiliations:** 1 Department of Community Health Sciences, Aga Khan University Karachi, Pakistan; 2 Genomic and Environmental Medicine, National Heart & Lung Institute, Imperial College London, United Kingdom; University of Global Health Equity, RWANDA

## Abstract

The construction industry in Pakistan employs 8% of the labour force, contributing 3% to the national GDP. This study aimed to determine the prevalence and associated factors of occupational injuries among construction workers in Karachi. We conducted a cross-sectional survey among 448 men from 10 construction sites in Karachi between June and October 2022. Workers aged ≥18 years involved in masonry, cleaning, machine operating, and supervision tasks were recruited and completed a structured, interviewer-administered questionnaire. Occupational injury was defined as “any injury at work over the last year for which the worker sought medical care or took time off work”. We found the prevalence of occupational injury to be 26% (n = 116). Workers from small construction were more likely to report injuries, with an OR of 2.01 (95% CI: 1.30–3.12). Additionally, those working more than 8 hours daily had greater odds of injury, with an OR of 2.30 (95% CI: 1.45–3.67). This study found a high prevalence of injuries among construction workers in Karachi, Pakistan, emphasizing the need for preventive measures and interventions for improving health and safety at construction sites.

## Background

The construction industry is a major employer, providing work to 7% of the global workforce, around 220 million people, and has an important impact on the economic development [1]. It generates about 15% of global GDP, and between 2016 and 2020 experienced a growth of approximately 2.5%, mainly in the Asia-Pacific region which now represents the largest construction market in the world [2,3]. Construction is positively associated with economic growth, especially in developing countries [4,5].

However, construction work is inherently dangerous [6]. Examples of hazardous tasks include excavation and work in confined spaces or at heights, operating power tools and heavy or electrical equipment, exerting force with the hands, repetitive movements, frequent or heavy lifting, pushing, pulling, or carrying heavy objects, working above head level, and maintaining prolonged, awkward postures [7]. The significant risks associated with these hazardous tasks and situations are reflected in a high frequency of injuries and fatalities, which are > 50% more common than in all other industries [8]. According to the International Labor Organization, the construction industry is responsible for more than half of all annual occupational injuries (270 million) and deaths (2 million) globally, resulting in disability, missed work and lost productivity [9]. However, there is a lack of reliable data on construction-related injuries in low- and middle-income countries (LMIC) due to limited safety awareness, inadequate equipment, poor reporting systems, and widespread informal employment [10].

Pakistan’s construction sector has experienced significant growth in recent decades, making it the second-largest sector in the country’s economy after agriculture [11]. Around 4.7 million, 8% of the labour force, are employed in the sector, with 35% of employment being directly or indirectly related to it, contributing 2.7% to the national GDP [12]. In Sindh province, it employs about 6% of the labour force [12]. There is a significant lack of data on the extent and causes of injuries among construction workers in the country, making it difficult to create effective intervention and education programs to enhance their safety and well-being. This survey aimed to determine the prevalence of occupational injuries among construction workers in Karachi, Pakistan, and to provide the information necessary to develop larger epidemiological studies that will support targeted strategies to improve health and safety in the sector.

## Methods

We undertook a cross-sectional survey across 10 construction sites in Karachi between June and October 2022. We included four small and six large sites based on the criteria described below. Karachi is the largest city and the principal seaport in Pakistan. Since the 1960’s it has grown rapidly with, now, an estimated population of above 25 million people [13]. The construction sites were selected purposively from companies registered with the Constructors Association of Pakistan (CAP) and the Government Labour Department, operating in different districts of Karachi (East, West, Central, South, and Malir). Due to feasibility concerns we could not undertake a proportionate sampling approach based on company sizes; workers were recruited based on availability at the time of field work. Except for a few instances, a similar number of workers were recruited across different sites (S1 Table). Workers at each site were identified with the help of human resources departments. Those engaged in active construction were present on the day of survey and were willing to participate were selected. Selected workers were approached by the data collectors for their consent. We excluded owners, managers, and other administrative personnel.

The sample size for this study was calculated using OpenEpi Info version 3.01, with a presumed injury prevalence of 38% [14] and a precision of 5%. Based on these inputs, the initial calculation yielded a sample size of 398 participants, consisting of 362 participants for the base sample size and an additional 36 (10%) to account for non-responses. However, several factors influenced the final sample size. These included the potential for missing data, variability in the available variables, and fluctuations in the number of construction workers present during data collection. To accommodate these considerations and ensure adequate statistical power, the final sample size was adjusted to 448 participants.

The main outcome variable was occupational injury which was a composite binary variable which included workers who had sustained any injury in the last year while doing construction work, and who had sought medical care for it or were consequently unable to carry out one or more work tasks due to the injury for a day or more [15].In case a worker had sustained more than one injury in the past year, only the most severe injury was considered for this analysis; subsequent questions were based on that specific injury to capture its severity and impact.

Co-variables included age, education, income, job title, educational status and size of the construction site. ‘Large’ sites accommodated multiple ongoing construction projects with high budgets, whereas ‘small’ sites covered smaller areas and employed fewer than 15 workers. Other variables included an employer safety practice score and a worker practice score. The employer safety practice score was a composite variable for health and safety training given to workers and provision of personal protective equipment (PPE) at the workplace; the worker practice score was a composite variable for the use of PPE in the workplace (uniform, facemask, goggles, gloves, safety shoes, protective hat); both variables were binary with categories of ‘good’ and ‘poor’ practice. Both practices scores were divided based on a percentage score, with a score of less than 50% classified as ‘poor’ practice and a score of 50% or more classified as ‘good’ practice Other important covariables included occupational details such as working hours per day, years of work in construction, type of work, and time and type of injury.

### Statistical analysis

We used STATA software version 17.0 for analysis. We reported all qualitative variables through their frequencies and proportions. For quantitative variables with normal distributions, we reported means and standard deviation; otherwise, median values and interquartile ranges (IQR) are presented. Following descriptive analysis, binary logistic regression was performed. In univariable analyses, we screened independent variables for potential inclusion in the multivariable model using a p-value cut-off of < 0.25, while statistical significance in the final multivariable model was determined using p < 0.05. We then checked for multicollinearity among the independent variables. High multicollinearity (cutoff: 0.8) was detected between employer safety practice and worker practice and between the duration of work and job title, leading to the removal of worker practice and duration of work from the model. All other independent variables with a p-value <0.25 in univariable analysis, or a very strong biological plausibility, were taken into multivariable logistic regression models. We reported odds ratios (OR) and 95% confidence intervals (95% CI), following multivariable analysis, p-value <0.05 was considered significant in the final model.

### Ethics

Ethical approval for the study was provided by the Aga Khan University Ethical Review Committee (ERC # 2022-6946-21145). Confidentiality of data and privacy of the participants was ensured throughout the data collection process. Both verbal and written formal consent was taken from construction workers. Workers were informed of the interviews a day beforehand, and thus interviews were conducted in a manner that did not affect the working schedule.

## Results

A total of 460 construction workers were approached for the study out of which 448 construction workers were enrolled and interviewed.

The median age of construction workers was 26 (21–36) years, and the median duration of employment in the construction sector, was seven years. A little over half of them (57%) had had no formal education (Table 1); 285 (64%) were working at large construction sites. Most 273, (61%) were machine operators and 115 (26%) workers worked for more than eight hours a day. Around 175 (39%) of the machine operators had no formal education, while 52 (12%) had middle or higher education; in contrast, only 16 (4%) supervisors had middle or higher education. Ninety-three (21%) workers had worked for 16 years or more, among whom 49 (11%) were machine operators and only nine (2%) were supervisors. The prevalence of occupational injuries was 116 (26%), as shown in Table 1. Frequency of occupational injuries was generally higher at small construction sites compared to large ones (S1 Table).

**Table 1:** Socio-demographic, occupation-related factors and occupational injury among construction workers (n = 448).

Variables	n (%)
Age in years^1^	26 (21-36)
Monthly income^1^ (PKR, in thousands^2^)	30 (24-42)
Size of construction site	
large	285 (63.6)
small	163 (36.4)
Educational status	
no formal education	253 (56.5)
primary	87 (19.4)
middle and above	108 (24.1)
Current smoker	81 (18.1)
Duration of employment (years)^1^	7 (3-15)
Job title	
machine operator/tool operator	273 (60.9)
mason	87 (19.4)
helper	63 (14.1)
supervisor	25 (5.6)
Main tasks at work^3^:	
hand demolition and clean up/surface and tuck-point grinding	204 (45.5)
concrete cutting and mixing	108 (24.1)
brick and concrete block cutting/block masonry work	102 (22.7)
others	34 (7.6)
Type of work according to risk of injury	
high	328 (73.2)
low	120 (26.8)
Working hours per day	
≤8	333 (74.3)
>8	115 (25.7)
Occupational injury^4^	116 (25.9)

^1^ *Median (interquartile range) reported for non-normally distributed variables.*

^2^ *280 Pakistani rupees ≈ $1 USD*

^3^ *Hand demolition and clean up, surface and tuck-point grinding, and brick and concrete block cutting/block masonry work are high-risk activities, whereas concrete cutting and mixing are considered low-risk activities* ^.^

^4^ *Occupational Injury = Workers who sustained any injury in last year while doing construction work, and in addition: sought medical care for injury or were unable to carry out task due to injury.*

Among those reporting an injury, the median number of injuries reported in the last year was 5 (2–9). In 83 (72%) workers the type of injury was a cut or laceration. According to the workers, in 91 (79%) of them the reason for injury was personal negligence, while in 36 (31%) it was due to a lack of supervision (**Table 2).**

**Table 2:** Site, type and causes of occupational injury among construction workers (n = 116).

Variables	n (%)
Body part injured	
lower limb/foot	50 (43.1%)
upper limb/hand	44 (37.9%)
head and neck/back/shoulders	22 (19.0)
Direct cause of injury	
struck by a falling, moving or stationary object	55 (47.4)
fall from height	31 (26.7)
strain from lifting, or bending, use of machinery/tools	30 (25.9)
Time of injury	
morning	75 (64.7%)
afternoon	41 (35.3%)
Type of injury	
cut/laceration	83 (71.6%)
muscle or ligament strain	17 (14.7%)
dislocation/fracture	10 (8.6%)
burn/electrocution	3 (2.6%)
eye injury	2 (1.7%)
internal injury	1 (0.9%)
Type of first aid	
bandage	31 (62.0)
cleaning/sutures	19 (38.0)
Self-treating after injury	
ice	1 (1.5)
heat	5 (7.6)
bed rest	2 (3.0)
over the counter medications	22 (33.3)
others	36 (54.5)
Restrictions due to injury	
none	52 (44.8)
daily living/occupational	64 (55.2)
Time off work due to injury	
None	26 (22.4)
<1 week	39 (33.6)
≥1 week	51 (43.9)
Effect of injury/illness:	
able to continue with all work duties	30 (25.9)
temporarily unable to do work duties	83 (71.5)
permanently unable to do some work duties	3 (2.6)
Reason for injury	
personal negligence	91 (78.5)
lack of safety equipment	60 (51.7)
lack of supervision	36 (31.0)
lack of worker occupational training	14 (12.1)
machinery malfunction	1 (0.9)

In relation to the employer safety practice scores, 167 (37%) of the workers reported ‘good’ scores, and 281 (63%) ‘poor’ scores. For the workers’ practice, 202 (45%) reported ‘good’ practice while 246 (55%) reported it to be ‘poor’ as shown in **Table 3**.

**Table 3:** Health and safety practices (n = 448).

Variables	n (%)
Employer safety practice^1^	
good	167 (37.3)
poor	281 (62.7)
Worker practice^2^	
good	202 (45.1)
poor	246 (54.9)
**Personal protective equipment (PPE) at work**	
Uniform	
always	78 (17.4)
sometimes	27 (6.0)
never	343 (76.6)
Face mask	
always	82 (18.3)
sometimes	63 (14.1)
never	303 (67.6)
Goggles	
always	70 (15.6)
sometimes	34 (7.6)
never	344 (76.8)
Gloves	
always	95 (21.2)
sometimes	65 (14.5)
never	288 (64.3)
Safety shoes	
always	197 (43.9)
sometimes	44 (9.8)
never	207(46.2)

^1^ *Employer safety practice is a composite variable for health and safety training for the workers and provision of PPE at the workplace.*

^2^ *Worker practice is a composite variable for the use of PPE at work (uniform, facemask, goggles, gloves, safety shoes, and protective cap)*

Among 163 workers at small construction sites, 134 (82%) had poor worker practice scores, and 138 workers (85%) reported poor employer safety practice scores. In contrast, among the 285 workers at large construction sites, 112 (39%) and 143 (50%) workers reported poor worker practices and poor employer safety practice scores respectively. Worker practice and employer safety practice were not strongly related to duration of employment in the construction sector. Among 134 workers with 1–3 years of experience, 90 (67%) reported poor employer safety practices scores; while among the 121 workers with 8–15 years of experience, this proportion was 63%. For worker practice, the corresponding proportions were 57% and 55%.

Among 116 construction workers who sustained an injury in the past year and as a consequence sought medical care or were unable to carry out tasks, 31 (27%) had been working in construction for 8–15 years, while 34 (31%) had been working in construction for 1–3 years and among them 64% of workers were machine operators.

**Table 4** presents factors that were statistically significant in univariable and multivariable logistic regression analyses. The independent predictors of occupational injury included working at small construction sites and working for more than 8 hours a day. While other variables such as job title, age, education, duration of employment and employer safety practices were not found to be statistically significant in this analysis.

**Table 4:** Crude and adjusted model for factors associated with occupational injury, among construction workers in Karachi (OR = odds ratio; CI = confidence interval).

Variables	Crude OR (95% CI)	Adjusted OR (95% CI)^a^ [*p-value*]
Size of the construction site		
large (reference)	1.00	1.00
small	1.96 (1.27-3.01) [0.002]	2.01 (1.30-3.12) [0.002]
Working hours		
< 8 hours (reference)	1.00	1.00
>8 hours	2.24 (1.42-3.55) [0.001]	2.30 (1.45-3.67) [<0.001]
Job title		
cleaner (reference)	1.00	1.00
machine operator	1.09 (0.58-2.04) [0.78]	–
mason	0.82 (0.38-1.75) [0.61]	–
supervisor	1.14 (0.40-3.23) [0.80]	–
Age		
18-21 years (reference)	1.00	1.00
22-26 years	0.84 (0.46-1.53) [0.58]	–
27-36 years	0.74 (0.41-1.33) [0.32]	–
37-60 years	1.01 (0.57-1.80) [0.96]	–
Employer safety practice		
good (reference)	1.00	1.00
poor	1.17 (0.75-1.83) [0.72]	–
Education		
middle & above (reference)	1.00	1.00
no formal education	1.28 (0.75-2.19) [0.35]	–
primary	1.33 (0.69-2.56) [0.38]	–

^a^ *The multivariable model was adjusted for size of the construction site, working hours, job title, age, income, employer safety practice and education.*

–empty cells represent variables that were dropped from the model as part of the stepwise regression process.

## Discussion

We undertook this survey to estimate the prevalence and associated factors of occupational injuries among construction workers in Karachi, Pakistan. In our study, the prevalence of occupational injury was 26% (116 workers), a proportion that was half to that among the construction workers in Gondar Town Ethiopia, in whom the prevalence of occupational injuries leading to their seeking medical care was around 50% [14]. Another study conducted by Yankson et al. among construction workers in Ghana showed that 9% of the workers were absent from work for 7–29 days, and 3% for more than 30 days, due to workplace injury in the past year [15]. Reasons for the variation in these findings include differences in injury definitions and in study settings.

Our findings suggest that the odds of occupational injury, is independently associated with working at small construction sites. A study conducted by Liao CW et al. among construction workers in Taiwan showed that the majority of occupational injuries occurred in small projects where most workers do not receive safety training despite engaging in high-risk work [16]. A cross-sectional survey of 673 construction workers in Norway suggests that this association is not confined to construction work in LMICs [17]. In our study among 163 workers at small construction sites, 138 (85%) reported poor employer safety practice. This is likely due to limited resources, lack of formal safety protocols, and reduced oversight at smaller sites, where safety measures are often not prioritized to favour cost and time efficiency [18]. Similarly, in our survey the odds of occupational injury were greater in construction workers who typically work shifts of > 8 hours. This observation is in line with a survey of Ethiopian construction workers among whom working for more than 48 hours per week was identified as a significant factor contributing to an increased risk of occupational injuries. Extended working hours can lead to physical and mental fatigue, reduced alertness, and impaired judgment, all of which increase the likelihood of accidents and injuries in the workplace [18].

Our study found that the odds of sustaining a workplace injury were not significantly associated with a worker’s job title. Although a notable proportion of injuries occurred among machine operators, this seems unsurprising considering the fact that a majority of this sample were machine operators. A study conducted among construction workers in Jeddah, Saudi Arabia, showed that accidents related to tools and material handling were more common leading to 23% of injuries being caused by hits from moving equipment or machinery and 25% of injuries resulting from material handling incidents, which often led to wounds, bleeding, fractures, and amputations [19]. Machine operators face a higher injury rate due to working with heavy, complex machinery that requires high concentration. Frequent exposure to hazards, physical strain, inadequate training, and poor safety measures further increases their risk of accidents [20].

Our results indicate that the odds of sustaining an occupational injury was not significantly associated with workers’ education status. This finding contrasts with the observations of Gebremeskel et al., who reported that Ethiopian construction workers with no formal education were three times more likely to experience occupational injuries [21]. Higher education may provide individuals with the skills and knowledge necessary to protect themselves from injury; as education levels increase, the incidence of unsafe actions tends to decrease. The higher rates of unsafe actions among individuals with low literacy may be attributed to their limited knowledge and awareness of safety practices. Additionally, workers with no formal education are often assigned more difficult and dangerous tasks, while educated workers are more likely to be employed in safer roles [22].

Nor, in our survey, was occupational injury significantly associated with poor employment practices which included health and safety training and the provision of PPE in the workplace. Nonetheless, 63% of the workers in our study reported a poor employer safety practice score. These findings contrast with a study by Lette et al. in Ethiopia, which demonstrated that the lack of safety training and PPE use increased the odds of injuries among construction workers by 5 and 3.6 times, respectively [23]. The absence of health and safety training and adequate PPE in the workplace significantly increases the risk of occupational injuries. Without proper training, workers may lack a clear understanding of potential hazards and the necessary safety protocols, leading to an increased likelihood of engaging in unsafe practices. Furthermore, when PPE is not provided or used correctly, workers are directly exposed to various dangers, such as harmful substances, sharp objects, or heavy machinery, which further heightens their vulnerability to serious injuries. This combination of insufficient training and inadequate protective measures creates a hazardous work environment that puts workers at greater risk.

These findings emphasize the need for robust workplace safety regulations and their strict enforcement, particularly in Karachi’s construction sector, where inadequate compliance continues to expose workers to significant occupational hazards. Workplace safety in Karachi’s construction sector is governed by the Occupational Safety and Health Code of Practice (OSH-CSP-2024) and the Sindh Occupational Safety and Health Act, 2017 [24]. The Pakistan Engineering Council (PEC), Sindh Labour Department, and Labour Inspectorates are responsible for monitoring construction safety, conducting inspections, and ensuring compliance. Large construction sites follow stricter regulations, requiring dedicated safety officers, PPE, training, and emergency preparedness, while small sites often lack proper enforcement and record-keeping. Challenges include weak enforcement, lack of worker awareness, and cost-cutting by small contractors. To improve implementation, authorities should increase inspections, mandate worker training, and offer compliance incentives to promote a safer construction environment in Karachi.

This survey has several strengths and provides a comprehensive assessment of occupational injuries among construction workers in Karachi, Pakistan, providing valuable data to inform future research and interventions. The inclusion of multiple construction sites, both large and small, ensured a diverse and representative sample. The relatively large sample size allowed for robust statistical analyses, providing estimates on the prevalence and associated factors of occupational injuries.

However, there are some limitations to consider. The cross-sectional design limits the ability to establish causality between the observed factors and occupational injuries, and our population may be one of relatively ‘healthy’ workers, underestimating the true burden of workplace injury in this sector. Reliance on self-reported data may introduce reporting and recall biases, particularly concerning the details and circumstances of injuries. The study’s focus on Karachi means the findings may not be fully generalizable to other regions of Pakistan or to other LMICs with different socio-economic and regulatory contexts. Furthermore, the impact of informal employment and undocumented work practices, which are prevalent in the construction industry, was not explored and could significantly influence injury rates and reporting.

## Conclusion

We found a high prevalence of injuries among construction workers in Karachi, Pakistan, emphasizing the urgent need for preventive measures and targeted interventions to improve health and safety at construction sites. The associations between injury prevalence and factors such as size of the site and working hours highlight areas needing focused attention. Implementing comprehensive safety training, enhancing PPE provision, and enforcing regulations to limit working hours could significantly reduce occupational injuries. Future research should include diverse regions, with studies of longitudinal design and should consider the impact of informal employment to develop more effective safety strategies.

## Supporting information

S1 Data Data on prevalence of injuries and factors associated with it among construction workers in Karachi, Pakistan. (XLSX)

S1 Table Frequency distribution of workers and occupational injuries across 10 construction sites in Karachi, Pakistan. (DOCX)
