# Supplementary material for: Prevalence of occupational injuries among construction workers in Karachi, Pakistan
Source: PLOS Glob Public Health. 2025 Apr 29;5(4):e0004578. doi: 10.1371/journal.pgph.0004578 (PMC12040193; doi:10.1371/journal.pgph.0004578)
Supplement: S1 Table — (DOCX) [file pgph.0004578.s002.docx]

**Supporting Information**

| **S1 table: Frequency distribution of workers and occupational injuries across 10 construction sites in Karachi, Pakistan** | | | |
| --- | --- | --- | --- |
| Construction sites | Type of construction site | Workers recruited from each site | Frequency of occupational injury^a^ |
|  |  | **n (%)** | **n (%)** |
| 1 | large | 51 (11.4) | 18 (35.3) |
| 2 | small | 48 (10.7) | 28 (58.3) |
| 3 | small | 50 (11.2) | 13 (26.0) |
| 4 | large | 50 (11.2) | 8 (16.0) |
| 5 | large | 50 (11.2) | 11 (22.0) |
| 6 | small | 34 (7.6) | 7 (20.6) |
| 7 | large | 53 (11.8) | 7 (13.2) |
| 8 | large | 51 (11.4) | 10 (19.6) |
| 9 | small | 31 (6.9) | 8 (25.8) |
| 10 | large | 30 (6.7) | 6 (20.0) |
| Total | (large; n=6)  (small; n=4) | 448 (100) | 116 (26) |
| *^a^Large construction sites (n=6) have more workers and a lower proportion of injuries, while small sites (n=4) have fewer workers but a higher injury rate* | | | |
